# Late-Stage Ovarian Cancer With Systemic Multiple Metastases Shows Marked Shrinkage Using a Combination of Wilms' Tumor Antigen 1 (WT1) Dendritic Cell Vaccine, Natural Killer (NK) Cell Therapy, and Nivolumab

**DOI:** 10.7759/cureus.56685

**Published:** 2024-03-22

**Authors:** Hisashi Nagai, Ryusuke Karube

**Affiliations:** 1 Human and Environmental Studies, Tokai University, Hiratsuka, JPN; 2 Oncology, Ginza Phoenix Clinic, Tokyo, JPN

**Keywords:** oncoimmunology, gynaecologic oncology, immune-checkpoint inhibitors, immuno-cell therapy, cancar immunotherapy

## Abstract

A patient with bilateral ovarian cancer, peritoneal dissemination, and multiple liver and lung metastases was found with a sudden accumulation of ascites six months after delivery. Chemotherapy was started, but the prognosis was judged to be poor, so immuno-cell therapy was combined with chemotherapy. After multiple cycles of Wilms' tumor antigen 1 (WT1) dendritic cell vaccine therapy and highly activated natural killer (NK) cell therapy, the patient showed a disappearance of ascites and a remarkable reduction of multiple cancers in the whole body. Furthermore, there were no side effects other than reactive fever caused by the administration of immune cells, and no damage to the patient's body was observed. This case suggests that not only the combined effects of chemotherapy and immunotherapy but also the combined use of various types of immuno-cell therapy may provide beneficial clinical effects in patients with extremely poor prognoses and few options for standard treatment.

## Introduction

Bilateral ovarian cancer with peritoneal dissemination and marked ascites is an extremely serious condition, for which chemotherapy is the only available treatment. Recently, the involvement of anti-tumor immune mechanisms has been reported as a mechanism of the anti-tumor effect of chemotherapy, and the synergistic effect of chemotherapy and immunotherapy is attracting attention [1,2].

Wilms' tumor antigen 1 (WT1) is a common cancer antigen that is highly expressed in various types of cancer, and its clinical usefulness has been highly evaluated by the National Cancer Institute study [3]. Although natural killer (NK) cell therapy has been attempted for its clinical application since the 1980s, NK cell therapy alone has not been as effective as expected [4].

In the case described in this paper, WT1 dendritic cell vaccine therapy (WT1-DC) was started simultaneously with chemotherapy, and multiple rounds of NK cell therapy were also administered concomitantly. Immuno-cell therapy, which has been thought to have low efficacy as a stand-alone therapy, may achieve high efficacy by combining these therapies, which was discussed based on this case.

## Case presentation

**Figure 1:**
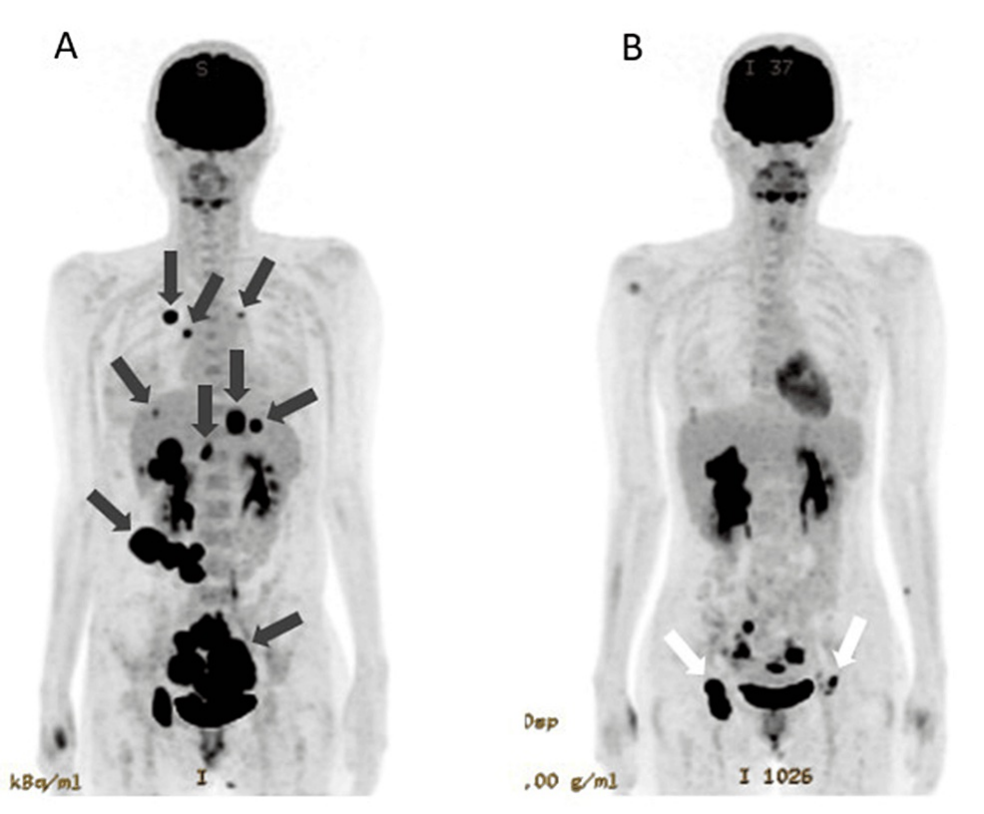
Marked shrinkage of cancer on PET-CT (A) Primary ovarian cancer and multiple metastases to the liver, peritoneum, and lungs are seen (black arrows). (B) PET-CT at 142 days shows marked shrinkage of the primary tumor, disappearance of lung metastases and peritoneal dissemination, and shrinkage of liver metastases. The white arrows indicate inflammatory reaction due to inoculation of dendritic cell vaccine to the inguinal region.

**Figure 2:**
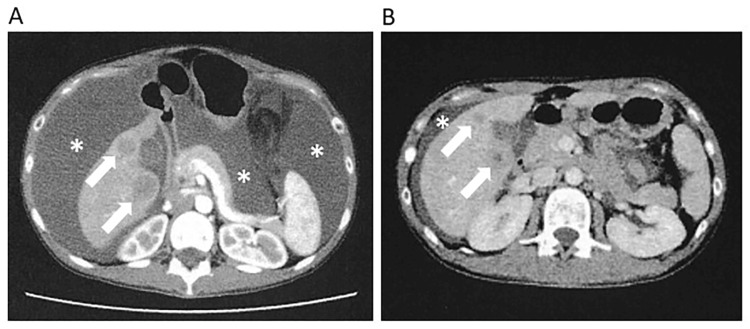
Marked reduction of ascites during NK cell therapy (A) Abdominal CT at diagnosis. A large amount of ascites is observed (*). White arrows indicate liver metastases. (B) Abdominal CT on the 56th day after diagnosis. Ascites was markedly reduced, and the size of liver metastases was also reduced.

A naturally healthy woman in her 30s experienced a sudden accumulation of ascites six months after the birth of her second child. Whole-body PET-CT 11 days before the definitive diagnosis revealed bilateral ovarian cancer, peritoneal dissemination, multiple liver metastases, bone metastases, and lung metastases, and she was diagnosed with Stage IV (Figure 1A). The pathological diagnosis was endometrial carcinoma. An abdominal CT scan on the day of the definitive diagnosis showed a significant amount of ascites (Figure 2A). Surgery and radiotherapy were not indicated, and paclitaxel 180 mg/m^2^ and carboplatin AUC5 were started every three weeks.

At the patient's request, immunotherapy was to be given concurrently with chemotherapy. WT1-DC and nivolumab 20 mg div were administered every two weeks starting on the 18th day. Monocytes collected via apheresis from the patient's peripheral blood were allowed to differentiate into mature DC over a two-week period at a cell culture processing facility. WT1 antigen was pulsed on the undifferentiated DC to complete the WT1-DC vaccine. One DC dose was adjusted to 27.4 x 10^6^ cells and administered in half doses near the bilateral inguinal lymph nodes.

**Figure 3:**
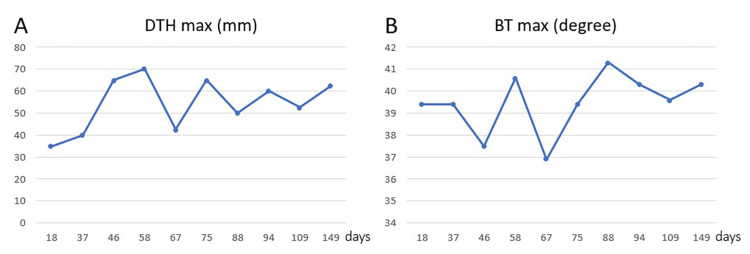
DTH and body temperature after WT1-DC administration A total of 10 times WT1-DC doses were administered every two weeks. (A) delayed-type hypersensitivity (DTH) max. The shortest and longest diameters of the erythema of the WT1 antigen inoculation sites were measured multiple times during the first three days after administration and averaged. The largest mean value of these was designated as DTH max. After the second inoculation, the diameter was over 40 mm, and the average value of all 10 inoculations was 54.3 mm. (B) Body temperature (BT) max. BT was measured multiple times during the three days after administration, and the highest of these temperatures was defined as BT max. Immunoreactivity was good, with a high fever of 39℃ or higher after the first administration.

**Figure 4:**
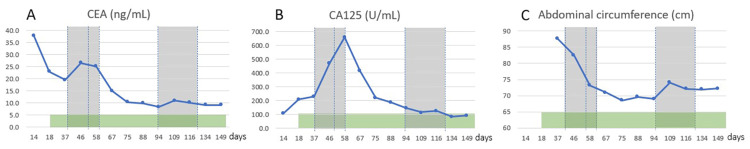
Transition of tumor markers and abdominal circumference length (A) Carcinoembryonic antigen (CEA), (B) carbohydrate antigen 125 (CA125), (C) abdominal circumference. CEA decreased temporarily with the start of chemotherapy, but began to rise on the 37th day. During three rounds of NK cell therapy, CEA and abdominal circumference decreased. CA125 continued to rise during the first three doses of NK, but decreased rapidly after the third dose. Green areas indicate the duration of WT1-DC vaccine therapy. Gray zones indicate the duration of NK cell therapy. The dotted line is the date of NK cell administration.

Additionally, 0.1 mL of WT1 was ingested on the medial side of the forearm, and the size of the appearing erythema (delayed-type hypersensitivity, DTH) was evaluated as an indicator of induction of WT1-specific cytotoxic T cells. A fever of 39.4℃ was observed on the night of the first administration, and the immune response was good with a DTH max of 35 mm within three days after administration (Figure 3). However, ascites was still evident on the 37th day, and while carcinoembryonic antigen (CEA) was decreased, CA125 was elevated. NK activity was measured to be low at an E/T ratio of 10:1 of 7.7% and 20:1 of 11.1%, so activated NK cell therapy was added (Figures 4A-4B).

NK cells were differentiated into highly activated NK cells from lymphocytes collected by peripheral blood collection for three weeks. Mature NK cells were administered on the 39th, 52nd, and 60th days; high fever over 40℃ was observed each time within three days after NK cell administration; after the third NK cell treatment, CEA and CA125 were markedly decreased, and abdominal circumference was also markedly decreased (Figure 4). Abdominal CT on the 56th day revealed a marked decrease in ascites and shrinkage of liver metastases compared to the time of diagnosis (Figure 2B).

Since the three times of NK cell treatments showed clinically beneficial improvement, additional NK cell treatments were given on days 94 and 123. With the fourth and fifth NK cell therapies, the mildly elevated CEA and ascites also began to decline again. On the other hand, CA125 continued to decrease continuously. The average number of cells per dose in NK cell therapy was 4.49 ± 2.2 x 10^9^, NK cell percentage was 56.9 ± 22.7%, NKT cell percentage was 20.7 ± 13.2%, T-cell percentage was 19.4 ± 10.6%, and live cell percentage was 93.4 ± 3.4%. PET-CT on day 142 showed that lung metastases and peritoneal dissemination had disappeared, and liver metastases and primary lesions were markedly reduced (Figure 1B).

**Figure 5:**
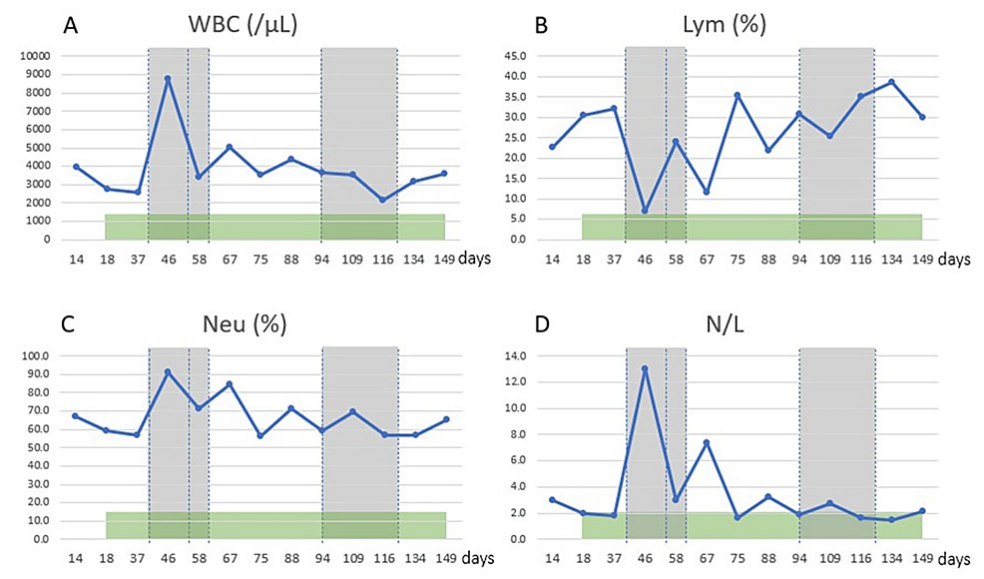
Transition of immune cells in blood (A) White blood cell, (B) percentage of lymphocytes in white blood cells, (C) percentage of neutrophils in white blood cells, and (D) neutrophil/lymphocyte ratio. The lymphocyte percentage increased, and the neutrophil percentage decreased during the course of immuno-cell therapy; the neutrophil/lymphocyte ratio gradually decreased, up and down, to a stable low value.

The neutrophil/lymphocyte ratio (N/L) in the blood continuously decreased during immunotherapy and shifted to lymphocyte predominance (Figure 5).

Due to the marked shrinkage of the tumor, the patient plans to consider radical resection of the primary tumor. The patient did not experience any side effects other than fever associated with the administration of dendritic cell vaccine or NK cells during the entire treatment period.

## Discussion

In this case, bilateral ovarian cancer with multiple metastases throughout the body was discovered six months after delivery due to a sudden accumulation of ascites fluid. The patient was started on chemotherapy only according to the standard treatment guidelines, but her doctor declared that the prognosis was poor, so the patient was started concomitant immuno-cell therapy.

WT1-DC vaccine was administered as the immuno-cell therapy. WT1 is one of the common cancer antigens, and according to a 2009 report by the National Cancer Institute in the US, WT1 ranked first among 75 common cancer antigens in terms of overall scores in nine categories, including therapeutic efficacy, immunogenicity, cancer specificity, and expression [3]. Lung adenocarcinoma also expresses WT1 in more than 90% of cases [5]. In the present case, WT1 was used as the target antigen of the dendritic cell vaccine, and WT1-DC administration was sufficient to induce WT1-specific cytotoxic T cells, as the patients showed high fever and a significant DTH averaging 54.3 mm in diameter [6,7].

CEA decreased after the initiation of DC vaccine therapy, while CA125 conversely increased. This indicates that the nature of the tumor changed with treatment. However, since ascites was still evident after the start of the dendritic cell vaccine and NK activity was low in blood tests, we decided to add highly activated NK cell therapy to the treatment in this case. The fact that the fever was higher than 40℃ each time the NK cells were administered suggests that the administered NK cells generated an immune response in the body. We have reported that fever induced by WT1 dendritic cell vaccine therapy is a marker of a good prognosis, and this kind of response is likely to be elicited in so-called “hot tumors” [7]. The same phenomenon may have occurred with NK cell therapy, especially the marked reduction in ascites, suggesting that peritoneal dissemination was markedly reduced by NK cell therapy.

The potential of NK cell therapy as a cancer immunotherapy has been investigated through preclinical studies since the 1980s [4]. It has been reported that patients with higher NK activity have longer progression-free survival [8], and it is now known that a certain level of clinical efficacy can be achieved by activating and proliferating NK cells ex vivo to significantly increase their antitumor activity. However, the therapeutic results of NK cells alone are not sufficient, and methods such as improvement of culture methods, genetic modification of NK cells, and combination with molecular targeted drugs are being explored [9,10].

Although there are many unknowns about the interaction between DCs and NK cells, it was already reported in Nature Medicine in 1999 that DCs activate NK cells by adhering to dormant NK cells in vivo, thereby promoting their anti-tumor effects [11]. DCs express TNF superfamily ligands on their membrane surface, which activate NK cells, and TNF from DC-derived exosomes is also known to activate NK cells by binding to the TNF receptor on NK cells [12].

It is also known that activated NK cells conversely activate DCs via secretion of INFγ and TNFα, and chemokines such as CCL4, CCL5, and XCL1 derived from NK cells also promote migration of DCs to the tumor site [13]. In short, there is no clear distinction between DCs as acquired immunity and NK cells as innate immunity, and it is possible that dense intercellular communication occurs between DCs and NK cells, which in turn determines the overall strength of anti-tumor immunity [14].

A febrile response after administration of immune cells is an indicator of anti-tumor immune activation in the patient's body. In the present case, a highly febrile response was observed after NK cell administration, but such a response is not seen in all patients. In our experience, when cancer patients are treated with NK cells alone, a febrile response is often absent (unpublished data). In this patient, the WT1-DC vaccine was initiated first, and NK cell therapy was added later. Considering the interaction relationship called the DC-NK axis, clinically meaningful anti-tumor effects may not be achieved by NK cell therapy alone but synergistically by the combination of NK cell therapy and DC vaccine therapy. Immune profile status (IPS) is a useful indicator of systemic anti-tumor immunity, and in this case, continuous improvement was observed during the course of WT1-DC vaccine therapy and NK cell therapy [15]. In particular, the N/L ratio, which was markedly elevated with a sharp rise in CA125, dropped sharply and stabilized at a low level after three rounds of NK cell therapy, suggesting that these treatments may be beneficial in extending life expectancy since an improvement in IPS is a useful indicator of long-term prognosis.

The combination of NK cell therapy with immune checkpoint inhibitors has recently been suggested to be a promising therapeutic strategy [16]. Similar to T cells, a variety of immune checkpoints have been found in NK cells, including PD-1, CTLA-4, TIM-3, and LAG-3, which work to suppress NK cell function through enhanced expression [17]. Antibody inhibition of PD-1 binding to PD-L1 is known to increase the antitumor activity of NK cells [18]. For example, the anti-PD-L1 antibody avelumab enhanced antibody-dependent cytotoxic activity through epigenetic priming of NK cells and tumor cells [19]. Studies have also shown that avelumab promoted NK cell cytokine synthesis and killed triple-negative breast cancer cells [20]. However, it is possible that NK cells are an innate immune system and regulate their function with immune checkpoints to prevent them from attacking autologous normal cells. Therefore, the combination therapy of NK cells and immune checkpoint inhibitors has a possibility to induce autoimmune reactions. Therefore, its efficacy and side effects need to be further verified.

## Conclusions

In this case, a combination of WT1-DC vaccine therapy, NK cell therapy, and nivolumab successfully reduced the size of Stage IV ovarian cancer with multiple systemic metastases markedly. There is an intercellular interaction between DCs and NK cells, called the DC-NK axis, which activates cellular functions on both sides and increases the anti-tumor effect. Even if the clinical efficacy of NK cell monotherapy is low, it may be possible to elicit high clinical efficacy when used in combination with a dendritic cell vaccine.

Since there are many unknowns in the communication between immune cells, there is a possibility that combined immunotherapy using multiple types of immune cells can produce a beneficial therapeutic effect. Future research on combined immunotherapy should be focused on.
